# Differential neutrophil gene expression in early bovine pregnancy

**DOI:** 10.1186/1477-7827-11-6

**Published:** 2013-02-05

**Authors:** Keiichiro Kizaki, Ayumi Shichijo-Kizaki, Tadashi Furusawa, Toru Takahashi, Misa Hosoe, Kazuyoshi Hashizume

**Affiliations:** 1 Laboratory of Veterinary Physiology, Department of Veterinary Medicine, Iwate University, Ueda 3-18-8, Morioka, Iwate, 020-8550, Japan; 2 Reproductive Biology Unit, National Institute of Agrobiological Sciences, Ikenodai 2, Tsukuba, 305-8602, Japan

**Keywords:** Pregnancy diagnosis, Neutrophils, Bovine, ISGs

## Abstract

**Background:**

In food production animals, especially cattle, the diagnosis of gestation is important because the timing of gestation directly affects the running of farms. Various methods have been used to detect gestation, but none of them are ideal because of problems with the timing of detection or the accuracy, simplicity, or cost of the method. A new method for detecting gestation, which involves assessing interferon-tau (IFNT)-stimulated gene expression in peripheral blood leukocytes (PBL), was recently proposed. PBL fractionation methods were used to examine whether the expression profiles of various PBL populations could be used as reliable diagnostic markers of bovine gestation.

**Methods:**

PBL were collected on days 0 (just before artificial insemination), 7, 14, 17, 21, and 28 of gestation. The gene expression levels of the PBL were assessed with microarray analysis and/or quantitative real-time reverse transcription (q) PCR. PBL fractions were collected by flow cytometry or density gradient cell separation using Histopaque 1083 or Ficoll-Conray solutions. The expression levels of four IFNT-stimulated genes, interferon-stimulated protein 15 kDa (*ISG15*), myxovirus-resistance (*MX*) *1* and *2*, and 2′-5′-oligoadenylate synthetase (*OAS1*), were then analyzed in each fraction through day 28 of gestation using qPCR.

**Results:**

Microarray analysis detected 72 and 28 genes in whole PBL that were significantly higher on days 14 and 21 of gestation, respectively, than on day 0. The upregulated genes included IFNT-stimulated genes. The expression levels of these genes increased with the progression of gestation until day 21. In flow cytometry experiments, on day 14 the expression levels of all of the genes were significantly higher in the granulocyte fraction than in the other fractions. Their expression gradually decreased through day 28 of gestation. Strong correlations were observed between the expression levels of the four genes in the granulocyte fractions obtained with flow cytometry and with density gradient separation.

**Conclusions:**

The expression profiles of *ISG15*, *MX1*, *MX2*, and *OAS1* could be a useful diagnostic biomarker of bovine gestation. Assessing the expression levels of these genes in a granulocyte fraction obtained with density gradient separation is a practical way of detecting gestation in cows within three weeks of insemination.

## Background

The early detection of gestation would help improve reproductive efficiency and bring economic benefits for the food animal industry [1,2]. Various effective methods for gestation confirmation have been developed, such as the detection of serum and/or milk progesterone levels [3-5], detection of pregnancy-associated glycoproteins [6], observation of estrous behavior, and the rectal or ultrasonic detection of the conceptus/corpus luteum [7,8].

Assessing the expression levels of interferon-tau (IFNτ)-stimulated genes (ISGs) in peripheral blood leukocytes (PBL) was recently proposed as a new approach to detecting gestation. IFNτ, which is used as a marker of pregnancy in ruminants, stimulates the expression of ISGs, such as interferon-stimulated protein 15 kDa (*ISG15*), interferon regulatory factors (*IRF*), and myxovirus-resistance (*MX*) proteins [9-15]. This new approach is generally derived from the molecular approach for analyzing the gene expression profiles of trophoblastic and endometrial tissues using microarray and quantitative real-time reverse transcription PCR (qPCR) in ruminants [14,16,17]. Although the PBL expression levels of some ISGs, such as *ISG15*, *MX1*, *MX2*, and 2′-5′-oligoadenylate synthetase (*OAS1*), have been found to be related to gestation [12,13,18], the most critical factor for gestation initiation remains to be clarified. In cows, there are reliable and useful methods (see above) for diagnosing gestation [3-8], and they have been applied in the field. However, diagnostic methods that produce results earlier, i.e., within about two weeks, are desired in order to shorten the open period. We hypothesized that particular PBL populations would display upregulated expression levels of genes related to pregnancy during early gestation in cows.

In the present study, we examined whether any PBL populations displayed upregulated expression levels of pregnancy-related genes during early gestation. First, the gene expression profiles of all PBLs were examined during early gestation using a custom-made bovine oligo microarray. Second, the PBLs were fractionated with flow cytometry or density gradient cell separation, and the gene expression profiles of each fraction were examined by qPCR. Finally, we searched for genes whose PBL expression levels could be used as specific markers of gestation establishment in cows.

## Methods

### Animals

The estrous cycles of Japanese Black x Holstein mixed cows (F1, multiparous) were synchronized via the administration of two intramuscular injections of prostaglandin F2α (0.15 mg Dalmazin, Kawasaki Pharmaceutical Co., Tokyo, Japan) every 14 days. Artificial insemination (AI) was performed on the day of estrus using frozen semen from Japanese black bulls. The day of AI was designated as day 0 of gestation, and blood was collected from the jugular vein in heparin sodium-containing vacutainers on days 0, 7, 14, 17, 21, and 28 of gestation. Blood was also collected before AI on day 0. Blood samples were used for microarray, qPCR, gradient, and flow cytometric analysis. The details of blood collection are summarized in Table 1. To analyze whole PBL, 19 cows were used. Fourteen cows were inseminated on day 0, and data were collected separately for the fertile (pregnant; *n* = 6) and infertile cows (not pregnant despite AI; *n* = 8). Blood samples were collected during the estrous cycle from the other cows as a control (not inseminated; *n* = 5). Only 5 out of 6 pregnant cows were used for microarray analysis. To analyze cells separated by flow cytometer or gradient, 10 cows were used. Data were collected separately for the pregnant (*n* = 5) and control cows (not inseminated, *n* = 3). Cells from these cows were used in both separation methods. The two other cows, inseminated but non-fertile, were not analyzed. Pregnancy was confirmed by transrectal ultrasonography (HS-1500 V, Honda Electronics, Aichi, Japan) at 4–5 weeks after AI. The guidelines for animal experiments outlined by the animal use and experimental committee of Iwate University (approval no.: 2007–46, 2008–59, and A201043), which approved this study, and the ethical guidelines of the National Institute of Agrobiological Sciences (approval no.: H18-036) were followed during the animal experiments.

**Table 1:** The number of samples (animals) used for each experiment

**Experiment**		**No. animals**	**Days after AI or estrous cycle**					
			**0**	**7**	**14**	**17**	**21**	**28**
Microarray P		5	5*	NC	3	NC	5	NC
qPCR	Non P	8	8	8	8	8	8	NC
	P	6	6	6	6	6	6	6
	EC	5	5	5	5	5	5	NC
FC and G	P	5	5	4	5	NC	4	4
	EC	3	3	3	3	NC	3	NC

*The number of samples used for analysis.

Blood samples for microarray analysis were picked up and used from the samples in pregnant cows of qPCR group. *NC*: not collected; *qPCR*: quantitative RT-PCR; *Non P*: non-pregnant; *P*: pregnant; *EC*: estrous cycle; *FC*: flow cytometric separation; *G*: gradient separation.

### Blood cell collection for microarray analysis

After blood collection, 9 mL of whole blood was carefully layered onto 6 mL of Histopaque-1119 (Sigma, Saint Louis, MO, USA) and centrifuged at 780 × *g* for 45 min at room temperature. The serum fraction was removed. Five hundred microliters of the upper part of the whole blood cell pellet fraction, which included red cells, was transferred into TRIzol LS (Invitrogen, Carlsbad, CA, USA) by pipette for total RNA extraction in accordance with the manufacturer’s protocol. DNase treatment to remove genomic DNA was performed using TURBO DNA-free kits (Ambion, Austin, TX, USA).

### Peripheral blood leukocyte (PBL) isolation for flow cytometry

After collection, the whole blood cell fraction was transferred to a new tube and suspended in lysis buffer (155 mM NH_4_Cl, 10 mM KHCO_3_, and 1 mM ethylenediaminetetraacetic acid) prepared at 37°C. The sample was then diluted with sorting buffer composed of Hank’s balanced salt solution (HBSS(−), Sigma) containing 2% fetal bovine serum and 10 mM 4-(2-hydroxyethyl)-1-piperazineethanesulfonic acid. The sample was inverted several times and immediately centrifuged at 1,200 × *g* for 10 min. The resultant pellets were resuspended in cold sorting buffer. Alternatively, some of the pellets were loaded onto Ficoll (Sigma)-Conray (Daiichi-Sankyo, Tokyo, Japan) solution (gravity: 1.072) for cell fractionation [19,20] and RNA extraction, as described below. Approximately 2 × 10^5^ cells were collected for the flow cytometric analysis (EPICS ALTRA MultiCOMP, Beckman Coulter, Carlsbad, CA, USA). The cells were stained with the following specific antibodies: anti-granulocyte antibody (2 μg/1 × 10^6^ cells, MM20A, VMRD Inc., Pullman, WA, USA), anti-bovine monocyte antibody (2 μg/1 × 10^6^ cells, BAQ151, VMRD Inc.), or anti-CD3 antibody (1 μg/1 × 10^6^ cells, MM1A, VMRD Inc.) for 60 min on ice. The cells were then washed and resuspended in sorting buffer containing Alexa488-conjugated anti-mouse IgG (0.05 μg/1 × 10^6^ cells, A-11001, Invitrogen). After 60 min of incubation on ice, the cells were washed, resuspended in staining buffer containing 5 μg/mL propidium iodide, and kept on ice until flow cytometry. Finally, we sorted the bovine PBL into granulocytes (G), monocytes (M), and lymphocytes (L) by flow cytometry based on the forward scatter channel (FSC) and the side scatter channel (SSC). The FSC roughly indicates a cell’s size, and the SSC shows the granularity of the cell. G display high FSC and SSC values, L display low FSC and SSC values, and M display intermediate FSC and SSC values. Based on these patterns, the PBL were sorted into four groups: all blood cells except dead cells (all), G, M, and L. Total RNA was extracted using the RNeasy Micro Kit (QIAGEN, Hilden, Germany) or the RNeasy Mini Kit (QIAGEN) with DNase treatment, in accordance with the manufacturer’s protocols, and used for the subsequent gene expression analysis.

### Microarray analysis

A custom-made 15 K bovine oligo DNA microarray developed at our laboratory was used for the microarray analysis (GPL9284), which was performed according to the method of a previous report [21]. After verifying the quality of the RNA with a 2100 Bioanalyzer (Agilent Technologies, Santa Clara, CA, USA) and NanoDrop ND-1000 spectrophotometer (NanoDrop Technology Inc., Wilmington, DE, USA), we performed one-color microarray analysis. RNA integrity was confirmed; all samples had an A260/280 ratio greater than 1.8 and an RNA integrity number greater than 8.0. The oligo-microarray produced by Agilent Technologies was used in this study. Sixty-mer nucleotide probes for the customized microarray were synthesized on a glass slide. cDNA synthesis, Cy3-labeled cRNA preparation, hybridization, and the washing and scanning of the array slides were performed according to the Agilent one-color microarray-based gene expression analysis protocol. Briefly, 400 ng of total RNA from each sample were reverse-transcribed into cDNA using the Quick Amp Labeling Kit (Agilent Technologies) with an oligo dT-based primer, and then Cy3-labelled cRNA was prepared by *in vitro* transcription. Labeled cRNA was purified with an RNeasy Mini Kit, and the concentration and Cy3 dye incorporation (pmol Cy3/μg cRNA) were measured with a spectrophotometer. Labeled cRNA (600 ng) was fragmented and hybridized using the Gene Expression Hybridization Kit (Agilent Technologies), according to the manufacturer’s instructions. The arrays were washed using a Gene Expression Wash Pack Kit and scanned using an Agilent Microarray Scanner (Agilent Technologies). Feature Extraction ver. 9.5 (Agilent Technologies) was used for image analysis and data extraction. The microarray data from each sample were imported into GeneSpring 12 (Agilent Technologies) for further data characterization. The GEO accession numbers are as follows. Platform: GPL9284; samples: GSM1052989 to GSM1053001; series: GSE42894.

### Quantitative real-time RT-PCR

The expression levels of *ISG15*, *MX1*, *MX2*, and *OAS1* were determined by qPCR according to the method described in a previous report [21]. Briefly, the DNase-treated total RNA (0.5 μg) was reverse-transcribed into cDNA using a High Capacity cDNA Reverse Transcription Kit (Applied Biosystems, Foster City, CA, USA) according to the manufacturer’s instructions. The primer pairs for the SYBR Green assay were designed using the Primer Express software (Applied Biosystems). The primers used to amplify each gene are listed in Table 2. Each qPCR reaction contained cDNA template, forward and reverse primers (300 nM each), and half volume of Power SYBR Green PCR Master Mix (Applied Biosystems) according to the manufacturer’s instruction. A non-template control was included. PCR and the resulting relative increase in reporter fluorescent dye emission were monitored in real time using an ABI7300 real-time PCR system (Applied Biosystems). The thermal cycling conditions included initial sample incubation at 50°C for 2 min and 95°C for 10 min, followed by 40 cycles of 95°C for 15 s and 60°C for 1 min. The relative differences in the initial amounts of each cDNA species were determined by comparing their threshold cycle (Ct) values. To quantify the mRNA copy number, standard curves for each gene were generated by serial dilution of the plasmid containing the corresponding cDNA. The dissociation curve for detecting the SYBR Green-based objective amplicon was confirmed because SYBR Green also detects double-stranded DNA, including primer dimers, contaminating DNA, and PCR products from misannealed primers. Contaminating DNA or primer dimers appear as a peak separate from the desired amplicon peak. The mRNA copy number of each gene was determined at each time point. In addition, the ratio of the mRNA expression level of each gene to that of glyceraldehyde-3-phosphate dehydrogenase (*GAPDH*) was also calculated to adjust for variations in the qPCR reaction. We analyzed other genes, such as ribosomal protein L27 and beta-actin, as other internal makers for qPCR, but there was no significant difference. All values are presented as mean ± standard error of the mean (SEM).

**Table 2:** Primer sequences for quantitative RT-PCR

**Gene**	**Accession no.**	**Forward primer**	**Reverse primer**
*ISG15*	NM_174366	GCAGACCAGTTCTGGCTGTCT	CCAGCGGGTGCTCATCAT
*MX1*	NM_173940	GAGGTGGACCCCCAAGGA	CCACCAGATCGGGCTTTGT
*MX2*	NM_173941	GGGCAGCGGAATCATCAC	CTCCCGCTTTGTCAGTTTCAG
*OAS1*	NM_178108	CCAAGTCAAACAAGCCATCGA	CACATCGGAAACACCTCTCCTT

### Granulocyte collection by gradient centrifugation

To collect the G fraction without using flow cytometry, whole blood samples (10 mL) were diluted with an equal volume of HBSS(−) and loaded onto Histopaque 1083 solution (Sigma) or Ficoll-Conray solution in a 50-mL conical tube (BD Falcon), then centrifuged at 1,000 × *g* for 30 min. After centrifugation, the upper (plasma) and middle (mononucleate cells) fractions were removed, then added 2 mL HBSS(−) and added 0.7% NH_4_Cl up to 50 mL. After a 5 min incubation on ice, the sample was centrifuged at 1,800 × *g* for 5 min, and the supernatant was removed. One milliliter of 0.7% NH_4_Cl was added to the tube, and the sample was incubated for 5 min on ice. The tube was centrifuged at 1,800 × *g* for 5 min. The resultant pellet was transferred to a new microtube (Eppendorf) for RNA extraction using TRIzol (Invitrogen) and gene analysis. In this experiment, blood samples were collected from ten cows: five pregnant cows (four subjected to blood sample collection throughout the experiment, one subjected to blood sample collection only on day 14) from days 0 to 28 of gestation, two non-pregnant cows (data were not included in the analysis), and three cyclic cows as a control.

### Statistical analyses

Microarray data were analyzed statistically with Student’s *t*-test and summarized using GeneSpring 12 (Agilent Technologies). All qPCR data and extracted microarray data were analyzed with one-way ANOVA followed by the Turkey-Kramer or Dunnett’s multiple comparison test using JMP 7 software (SAS Institute Inc., Cary, NC, USA). The correlations between the data in microarray and qPCR were analyzed with the Spearman’s rank correlation analysis (correlation coefficient: *rs*) using JMP software because the data were non-parametric. The correlations between the flow cytometric collection and gradient collection were determined by the Pearson product–moment correlation coefficient using JMP software, and the correlation coefficients were calculated as *r*-values.

## Results

### Global gene expression profiles of PBL

Gene expression in PBL during early gestation was assessed using an oligo-microarray. On days 14 and 21, the expression of 72 and 28 genes, respectively, was significantly increased, compared with expression on day 0 (see Additional file 1: Table S1 and Additional file 2: Table S2). Some genes that respond to IFNτ (*ISG15*, and *MX2*) were only significantly upregulated on day 21.

**Figure 1:**
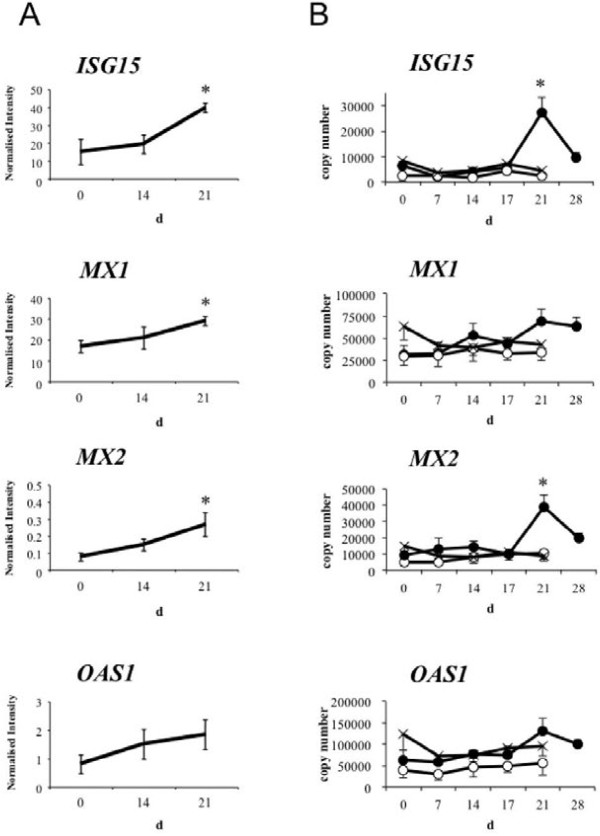
**Microarray (A) and qPCR (B) analysis of IFNτ-stimulated gene expression in PBL.** (**A**) Blood was collected from cows on days 0 (*n* = 5), 14 (*n* = 3), and 21 (*n* = 5) after artificial insemination (AI). Three and five cows samples were anlyzed on day 14 and 21 of gestation, respectively. Each sample was applied to an oligo microarray. Data are shown as mean ± SEM. (**B**) Open circles represent estrous cycle cows (control, *n* = 5). Crosses and solid circles represent infertile cows (*n* = 8) and pregnant cows (*n* = 6), respectively, which were subjected to AI on day 0 and diagnosed via ultrasonography. Data are shown as mean ± SEM. The copy number of each mRNA is indicated to confirm the minimum amount of mRNA produced. Values significantly different from the value on day 0 of gestation (before AI) are shown with an asterisk. P values less than 0.05 were considered significant. d: day of gestation and/or estrous cycle.

Microarray analysis showed that in the whole PBL population the expression levels of *ISG15*, *MX1*, *MX2*, and *OAS1* increased as gestation progressed (Figure 1). qPCR confirmed the expression of the four ISGs in whole PBL; the copy numbers of *ISG15* and *MX2* were significantly higher on day 21 of gestation than on day 0 of gestation, and *MX1* and *OAS1* were increased but not significant (Figure 1B). However, when examined the expression levels of some of the genes in Additional file 1: Tables S1 and Additional file 2: Table S2 (see Additional files 1 and 2) except abovementioned ISGs more closely, we found that there were no common significant genes (over 2 fold changes) between days 14 and 21 of gestation except three geens; FERM domain containing 4A (NM_001192267), low density lipoprotein receptor-related protein 1 (XR_082763) and EST (EE896098), by microarray. The microarray gene expression data and qPCR data showed a strong correlation for *ISG15* (*rs* = 0.709, p = 0.0067), *MX1* (*rs* = 0.571, p = 0.0413), *MX2* (*rs* = 0.692, p = 0.0087), and *OAS1* (*rs* = 0.923, p < 0.001).

### Fractionation of peripheral blood leukocytes and the gene expression levels of each fraction

**Figure 2:**
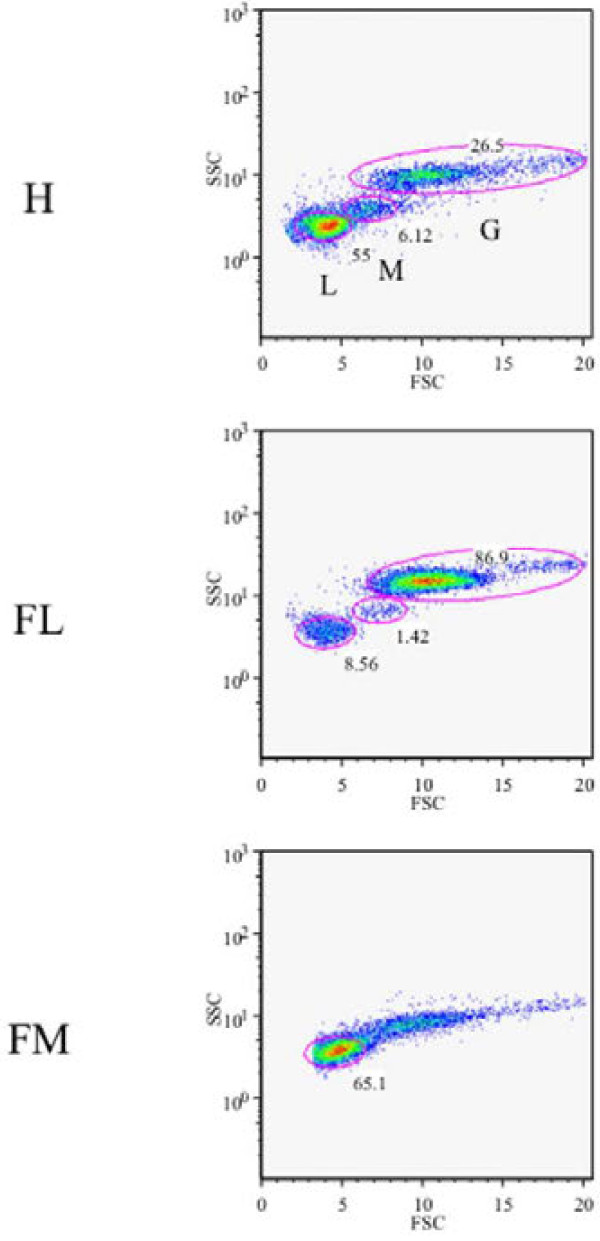
**Characteristics of cells in the Ficoll-Conray fractions.** After obtaining cell fractions with the Histopaque 1119 and Ficoll-Conray gradient methods, the characteristics of the cells and their proportions were determined by flow cytometry using a range of cell collection gates (SSC and FSC). H: whole PBL obtained with Histopaque 1119; FL: lower fraction obtained with Ficoll-Conray; FM: middle fraction obtained with Ficoll-Conray. L: lymphocytes; M: monocytes; G: granulocytes. The numbers indicate the frequency of each cell type. Representative figures are shown.

**Figure 3:**
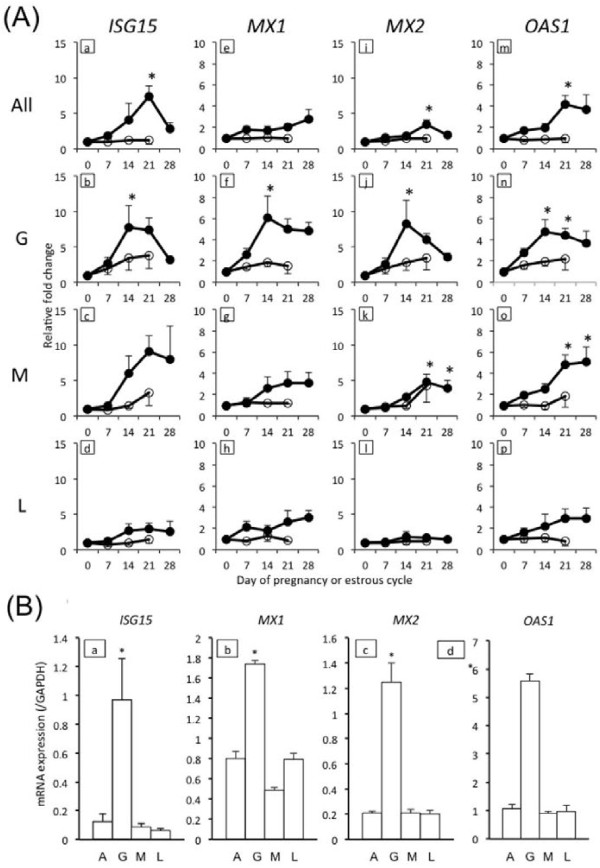
**mRNA profiles of each PBL fraction.** (**A**) Profiles of *ISG15* (a to d), *MX1* (e to h), *MX2* (i to l), and *OAS1* (m to p) mRNA levels in the four PBL fractions: all blood cells except dead cells (all), granulocytes (G), monocytes (M), and lymphocytes (L) in early pregnant (closed circles; *n* = 4) and cyclic (open circles; *n* = 3) cows. Values were adjusted for *GAPDH* mRNA expression. They represent the relative fold change compared with the value on day 0. The error bars represent the SEM. Values significantly different from the value on day 0 of gestation are shown with an asterisk. P values less than 0.05 were considered significant. (**B**) *ISG15* (a), *MX1* (b), *MX2* (c), and *OAS1* (d) mRNA levels in the PBL fractions on day 14 of pregnancy (*n* = 5). A: all fractions; G: G fraction; M: M fraction; L: L fraction. Values represent the mean values adjusted for *GAPDH* mRNA expression, and the error bars represent the SEM. Values significantly different from the value in all fractions are shown with an asterisk (p < 0.05).

We hypothesized that the expression levels of genes involved in gestation would vary between different types of PBL. Therefore, the PBL were fractionated using the Ficoll-Conray method, and the fractions were subjected to flow cytometry. The specificity of each fraction was confirmed with flow cytometric analysis using antibodies specific for different PBL cell types (see Additional file 3: Figure S1). Thereafter, cells were identified as G, M, and L according to their FSC and SSC scatter plot profiles without using antibodies (Figure 2) were analyzed in each fraction until day 28 of gestation using qPCR (Figure 3). On day 14, all four genes displayed significantly higher expression levels (p < 0.05) in the G fraction than in the other fractions, including the non-separated blood cells (all, Figure 3). After day 14, the expression of *IGS15*, *MX1*, *MX2*, and *OAS1* in the G fraction gradually decreased through day 28 of gestation.

### Simplification of the granulocyte collection method

When we separated the whole blood cell population into three fractions (upper, middle, and lower fractions) using Ficoll-Conray or Histopaque solutions, we found about 90% of the G in the lower fraction. The accuracy of our G sorting method was confirmed by flow cytometry using anti-bovine G antibody and morphological analysis. The gene expression levels of the four target genes in the G fractions obtained with flow cytometry and with Ficoll-Conray separation displayed high correlation coefficients (see Additional file 4: Figure S2); the correlation coefficients (*r*) for *ISG15*, *MX1*, *MX2*, and *OAS1* were 0.892 (p = 0.0070), 0.883 (p = 0.0084), 0.893 (p = 0.0068), and 0.822 (p = 0.0232), respectively.

## Discussion

ISGs were detected in PBL, and their expression pattern correlated with the onset of gestation. Our results confirm those from previous studies [12-15]. The present study showed G fraction was a specific expression PBL cell for ISGs even though other PBL cells expressed ISGs. ISG expression in G may be a suitable diagnostic indicator of early gestation in cows. Methods for the early diagnosis of pregnancy in cattle have been sought because such methods will improve the reproductive and running efficiency of the cattle industry [1,2]. Although many technologies can diagnose gestation, including rectal palpation, ultrasonography, and milk progesterone tests, these methods are generally applicable three weeks after fertilization [3-8]. The present study describes a new method for determining pregnancy within three weeks, specifically 14–21 days after AI. This method assesses the expression of ISGs in the G fraction of PBL using gradient separation and qPCR. The technique is more specific and practical than the traditional methods of gestation diagnosis mentioned above.

Microarray analysis is a very useful method for assessing gene expression profiles, and it has become a popular method for examining physiological events in food production animals [16,17,22-24]. However, recent studies have shown that while microarray analysis is a useful method for determining the gene expression profiles of cattle reproductive tissues, it might be impractical for the routine diagnosis of gestation [12-14]. Although we previously described some preliminary results on ISG expression obtained using PBL, the expression of ISGs varied widely [14]. Green et al. [13] carefully examined whether it was possible to use the gene expression profile of leukocytes for gestation diagnosis and concluded that assessing the expression levels of ISGs is a reliable method for pregnancy detection, especially in heifers. In the present study, only multiparous cows were used, but ISG expression appeared to be a suitable indicator. Another group confirmed that the expression of ISGs in peripheral blood mononuclear cells reflects embryonic mortality during early gestation [18]. These findings generally agree with those obtained in the present study, in which ISG expression was more reliable in the G fraction. In addition, microarray analyses in the study by Green et al. [13] and in the present study detected various genes related to gestation events, but the microarray expression profiles obtained in the two studies differed. Differences between strains and in the parous status of the cows may account for the discrepancies.

The PBL gene expression profile is considered a potential source of markers that reflect the physiological changes that occur in early gestation because PBL are involved in various immunological responses. Recent transcriptome analysis studies have shown that PBL are an excellent source of biological markers of physiological conditions in humans and other animals [25-27]. For example, gene expression profiles display extensive, rapid, and complex changes that reflect the changes in the levels of active humoral substances in the circulation, such as those caused by infection, inflammation, cytokines, and hormones [28,29]. Thus, PBL gene expression profiles might be a good source of biomarkers of changes of physiological significance [15].

We analyzed PBL to identify the cell subset that is most sensitive to gestation signals from the conceptus in mother cows. The most important issue is the development of an accurate and simple method for collecting target blood cells. Consistent with previous studies, microarray data suggest that various genes could be used as biomarkers of pregnancy, including ISGs [13,21-24,30-32]. IFNτ stimulates various genes in the bovine endometrium, and some of them are expressed in PBL [12-15]. The endometrial response to the presence of a fertilized embryo or the conceptus is surprisingly rapid, and previous studies have suggested that such stimuli affect endometrial cells and blood cells. In the present study, we selected *ISG15*, *MX1*, *MX2*, and *OAS1* as potential markers of gestation. The expression levels of these genes increased from days 14 to 21 after AI, but declined by day 28. The changes in the expression levels of these genes might be related to changes in the concentration of IFNτ during gestation [33]. *ISG15*, *MX1*, *MX2*, and *OAS1* displayed significantly increased expression levels in the G fraction, especially from days 14 to 21 of gestation, and displayed less significant increases in their expression levels in the whole blood cell population.

The reason and mechanism for the specific stimulation of G by IFNτ are unclear because G have short lives (less than a week) in the peripheral blood. In addition, the increases in the expression levels of *ISG15*, *MX1*, *MX2*, and *OAS1* started between days 7 to 14 of gestation, when the bovine conceptus has not begun producing IFNτ [33]. A low level of IFNτ may be sufficient to enhance these genes. Interestingly, IFNτ is a 23-kDa protein that is only produced by the conceptus, which secretes it into the uterus. How then does IFNτ reach blood cells? There is clear evidence that IFNτ passes into the blood via the uterine vein [10,34], though a detailed study is needed examine whether this large molecule is able to pass from the uterine cavity to the peripheral blood stream. Another question is whether the expression of ISGs is induced by IFNτ or viral infection. ISGs respond to signals from the conceptus and from viruses, such as the bovine viral diarrhea virus (BVDV), which causes infectious diarrhea and mucosal disease in calves [35,36]. It is difficult to eliminate the effects of BVDV infection on the expression of ISGs following the start of conception. However, G have a short life in the blood stream, perhaps less than a week, and thus they may quickly and specifically respond to gestational signals from the conceptus. In fact, IFNτ signals through the *Janus* family of tyrosine kinases (JAK) and the signal transducer and activator of transcription (STAT) pathway [37]. The phosphorylation of STAT1 is a critical step in the cell signaling response to type I interferons, including IFNτ. The phosphorylation intensity of STAT1 is different in various peripheral blood white cells; the intensity in L is higher than that in G [38]. These results suggest a possible way to establish whether the signal cascade derives from BVDV because the expression intensities of ISGs were specifically higher in G than in L in the present study. This may mean that the responsiveness and sensitivity of PBL to the ISG signaling pathway depends on the cell type. Therefore, ISG expression in the G fraction may be a possible indicator for early gestation diagnosis in cattle, but it does not provide any direct evidence about how IFNτ stimulates the expression of ISGs in circulating blood cells, specifically G. Further examinations are required to clarify the sensitivity of G to viral infections and gestation signals, mainly IFNτ. We also detected more than 20 new genes other than ISGs that increased after the onset of gestation (Additional file 1: Table S1 and Additional file 2: Table S2). They may be new indicator genes. Further studies are necessary to find new makers.

Flow cytometric analysis showed that the main IFNτ-responsive blood cell during early gestation in bovines was G. This is the first evidence that G specifically respond to gestation signals, including IFNτ. However, although cell fractionation with a flow cytometer is an accurate method, it is not practical for everyday use. A simpler and more convenient method is required for routine use. Thus, we used two density gradient solutions, Ficoll-Conray and Histopaque, as a cell collection method. Both solutions worked well. The accuracy of these methods was confirmed by morphological examinations using Giemsa staining and flow cytometry. Gradient separation is an accurate and simple way to isolate PBL, especially the G fraction.

## Conclusions

Flow cytometric separation of PBL suggested that G are most sensitive to IFNτ stimulation, and that the expression levels of ISGs in the G fraction might be good biomarkers for diagnosing pregnancy in bovines very early during gestation. The density gradient cell method for the collection of PBL is a useful way of identifying G. It represents a simpler and more practical method for diagnosing bovine pregnancy in cows within three weeks of insemination.

## Abbreviations

BVDV: Bovine viral diarrhea virus; G: Granulocytes; IFN: Interferon; ISG: Interferon-stimulated gene; L: Lymphocytes; M: Monocytes; MX: Myxovirus-resistance; qPCR: Quantitative real-time reverse transcription PCR; OAS1: 2′-5′-oligoadenylate synthetase; PBL: Peripheral blood leukocytes; STAT: Signal transducer and activator of transcription.

## Competing interests

The authors declare that they have no competing interests.

## Authors’ contribution

KK collected samples, conducted microarray, planned the study, and drafted the manuscript. AS-K collected samples and conducted microarray, flow cytometric analysis, and qRT-PCR. TF carried out flow cytometric analysis. TT and MH collected samples and prepared animals. KH designed the experiments, prepared samples, and wrote the manuscript. All authors read and approved the final manuscript.

## Supplementary Material

Additional file 1 **Table S1.** Genes whose expression increased significantly between D0 and D14 of gestation. Fold change from D0 to D14 assessed by microarray analysis (*n* = 3). Click here for file

Additional file 2 **Table S2.** Genes whose expression increased significantly between D0 and D21 of gestation. Fold change from D0 to D21 assessed by microarray analysis (*n* = 5). Click here for file

Additional file 3 **Figure S1.** Separation of blood cells with specific antibodies using flow cytometry. H: whole blood white cells; FL: lower fraction obtained with Ficoll-Conray separation; FM: middle fraction obtained with Ficoll-Conray separation; NS: no stain; IgG: collected with IgG; G: collected with anti-granulocyte antibody; M7: collected with anti-monocyte antibody; CD3: collected with anti-CD3 antibody. The numbers indicate the percentage of cells in each antibody-derived fraction. They are the mean of four independent examinations (*n* = 4). Representative figures are shown. Click here for file

Additional file 4 **Figure S2.** Correlations between gene expression profiles in granulocyte fractions obtained by flow cytometry and Ficoll-Conray separation. Blood samples were divided and simultaneously analyzed using the two different methods. G: granulocyte, FL: flow cytometry. Data were normalized to *GAPDH* expression (*n* = 7). Click here for file
