# Supplementary material for: Differential neutrophil gene expression in early bovine pregnancy
Source: Reprod Biol Endocrinol. 2013 Feb 5;11:6. doi: 10.1186/1477-7827-11-6 (PMC3570308; doi:10.1186/1477-7827-11-6)
Supplement: Additional file 1 — Table S1. Genes whose expression increased significantly between D0 and D14 of gestation. Fold change from D0 to D14 assessed by microarray analysis (n = 3). [file 1477-7827-11-6-S1.docx]

**Additional file 1: Table S1 - Genes whose expression increased significantly between D 0 and D14 of gestation**

Fold change from D 0 to D 14 assessed by microarray analysis (*n* = 3).

| Accession No. | Fold change | Description |
| --- | --- | --- |
| BP101882 | 32.76 | Transcribed locus |
| XM_002686122 | 5.38 | prostaglandin F2 receptor negative regulator |
| BP103217 | 4.90 | Transcribed locus |
| BM481136 | 4.73 | Transcribed locus |
| NM_174109 | 3.46 | melanocortin 2 receptor (adrenocorticotropic hormone) |
| XM_001787381 | 3.44 | l(3)mbt-like 1 (Drosophila) |
| AW314082 | 3.41 | Transcribed locus |
| BP107193 | 3.37 | Transcribed locus |
| XM_612075 | 3.05 | LIM domain only 3 (rhombotin-like 2) |
| NM_001102506 | 3.02 | protocadherin alpha 13 |
| NM_001038126 | 2.92 | solute carrier family 43, member 3 |
| NM_174196 | 2.89 | thrombospondin 1 |
| NM_001015538 | 2.81 | cyclin Pas1/PHO80 domain containing 1 |
| BE685181 | 2.76 | Transcribed locus |
| XM_002691147 | 2.76 | AF4/FMR2 family, member 3 |
| NM_001192516 | 2.76 | sprouty-related, EVH1 domain containing 1 |
| BP108106 | 2.74 | Transcribed locus |
| NM_174262 | 2.71 | cholecystokinin B receptor |
| NM_178318 | 2.71 | biglycan |
| BP102657 | 2.71 | Transcribed locus |
| NM_174052 | 2.68 | fetal and adult testis expressed 1 |
| NM_001192267 | 2.67 | FERM domain containing 4A |
| NM_001105343 | 2.64 | ATP-binding cassette, sub-family G (WHITE), member 4 |
| CB443052 | 2.62 | Transcribed locus |
| NM_001081517 | 2.59 | chromosome 1 open reading frame 210 ortholog |
| BI538589 | 2.59 | Transcribed locus |
| XM_001256807 | 2.59 | FLJ00021 protein-like |
| AF451170 | 2.59 | Transcribed locus |
| NM_001102478 | 2.57 | nerve growth factor receptor |
| XM_581497 | 2.48 | Transcribed locus |
| NM_001076417 | 2.48 | endoplasmic reticulum-golgi intermediate compartment (ERGIC) 1 |
| BP103565 | 2.46 | Transcribed locus |
| CB464215 | 2.44 | Transcribed locus |
| BE664601 | 2.43 | Transcribed locus |
| NM_182788 | 2.41 | tissue factor pathway inhibitor 2 |
| AW357744 | 2.40 | Transcribed locus |
| BP113016 | 2.39 | Transcribed locus |
| NM_001101155 | 2.37 | nidogen 1 |
| NM_001192894 | 2.36 | calpain 5 |
| CB435807 | 2.35 | Transcribed locus |
| BP110226 | 2.35 | Transcribed locus |
| XM_001788161 | 2.33 | utrophin |
| XM_002684818 | 2.33 | mucin 20, cell surface associated |
| NM_001031753 | 2.33 | chromosome 12 open reading frame 43 ortholog |
| XM_615785 | 2.31 | protein tyrosine phosphatase, receptor type, U |
| XM_002688847 | 2.30 | erythropoietin receptor |
| BP106734 | 2.29 | Transcribed locus |
| AW426007 | 2.26 | Transcribed locus |
| CB457387 | 2.25 | Transcribed locus |
| NM_001035400 | 2.22 | arylsulfatase family, member K |
| XR_082763 | 2.22 | low density lipoprotein receptor-related protein 1 |
| NM_001038095 | 2.22 | amyloid beta (A4) precursor-like protein 1 |
| XM_002688904 | 2.18 | SH2 domain containing 3A |
| CB227343 | 2.17 | Transcribed locus |
| NM_001083478 | 2.16 | EGF-like, fibronectin type III and laminin G domains |
| NM_001001135 | 2.16 | collagen, type II, alpha 1 |
| BP112439 | 2.15 | Transcribed locus |
| NM_001075583 | 2.15 | G protein-coupled receptor 4 |
| NM_001077115 | 2.13 | aminoacylase 1 |
| BI538319 | 2.12 | Transcribed locus |
| NM_001046434 | 2.12 | epsin 3 |
| NM_001105330 | 2.12 | chymotrypsinogen B1 |
| CB457414 | 2.10 | Transcribed locus |
| BP102161 | 2.09 | Transcribed locus |
| BI539215 | 2.09 | Transcribed locus |
| NM_174008 | 2.07 | CD14 molecule |
| XM_002700247 | 2.06 | nuclear receptor subfamily 0, group B, member 1 |
| NM_001075978 | 2.06 | D-2-hydroxyglutarate dehydrogenase |
| NM_001075592 | 2.05 | MANSC domain containing 1 |
| BI535273 | 2.03 | Transcribed locus |
| EE896098 | 2.01 | Transcribed locus |
| BP111686 | 2.00 | Transcribed locus |
