# Supplementary material for: Differential neutrophil gene expression in early bovine pregnancy
Source: Reprod Biol Endocrinol. 2013 Feb 5;11:6. doi: 10.1186/1477-7827-11-6 (PMC3570308; doi:10.1186/1477-7827-11-6)
Supplement: Additional file 2 — Table S2. Genes whose expression increased significantly between D0 and D21 of gestation. Fold change from D0 to D21 assessed by microarray analysis (n = 5). [file 1477-7827-11-6-S2.docx]

**Additional file 2: Table S2 - Genes whose expression increased significantly between D0 and D21 of gestation**

Fold change from D0 to D21 assessed by microarray analysis (*n* = 5).

| Accession No. | Fold change | Description |
| --- | --- | --- |
| NM_001024557 | 7.31 | 2'-5'-oligoadenylate synthetase 2, 69/71kDa |
| NM_174007 | 7.20 | chemokine (C-C motif) ligand 8 |
| NM_174366 | 4.44 | ISG15 ubiquitin-like modifier |
| NM_001192267 | 4.22 | FERM domain containing 4A |
| BP101516 | 4.06 | Transcribed locus |
| NM_001034492 | 3.88 | complement component 2 |
| BM446421 | 3.83 | Transcribed locus |
| NM_173941 | 3.80 | myxovirus (influenza virus) resistance 2 (mouse) |
| BF604728 | 3.78 | Transcribed locus |
| XR_082763 | 3.41 | low density lipoprotein receptor-related protein 1 |
| CB467307 | 3.13 | Transcribed locus |
| BE682247 | 2.59 | Transcribed locus |
| XM_002695068 | 2.45 | AXL receptor tyrosine kinase |
| NM_001079646 | 2.43 | FXYD domain containing ion transport regulator 3 |
| XM_001789172 | 2.43 | plexin D1 |
| BE683736 | 2.41 | Transcribed locus |
| BF440504 | 2.41 | Transcribed locus |
| NM_001045941 | 2.33 | radical S-adenosyl methionine domain containing 2 |
| BM285701 | 2.25 | Transcribed locus |
| XM_002699405 | 2.25 | low density lipoprotein receptor-related protein 5 |
| EE896098 | 2.11 | Transcribed locus |
| NM_001015545 | 2.09 | RNA helicase LGP2 |
| CB171863 | 2.09 | Transcribed locus |
| BI534945 | 2.07 | Transcribed locus |
| NM_001034399 | 2.05 | FXYD domain containing ion transport regulator 6 |
| NM_001081581 | 2.03 | 4-aminobutyrate aminotransferase |
| CB426963 | 2.03 | Transcribed locus |
| NM_001144088 | 2.02 | SH3-domain binding protein 4 |
