# Supplementary figures and images for: Differential neutrophil gene expression in early bovine pregnancy
Source: Reprod Biol Endocrinol. 2013 Feb 5;11:6. doi: 10.1186/1477-7827-11-6 (PMC3570308; doi:10.1186/1477-7827-11-6)

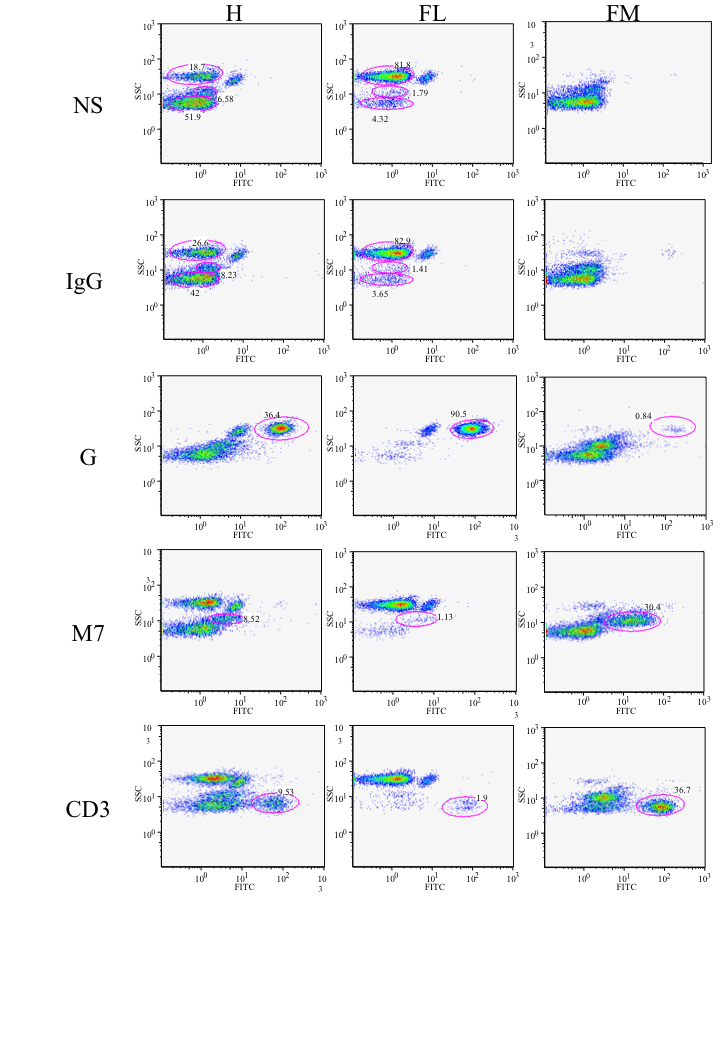

Supplement: Additional file 3 — Figure S1. Separation of blood cells with specific antibodies using flow cytometry. H: whole blood white cells; FL: lower fraction obtained with Ficoll-Conray separation; FM: middle fraction obtained with Ficoll-Conray separation; NS: no stain; IgG: collected with IgG; G: collected with anti-granulocyte antibody; M7: collected with anti-monocyte antibody; CD3: collected with anti-CD3 antibody. The numbers indicate the percentage of cells in each antibody-derived fraction. They are the mean of four independent examinations (n = 4). Representative figures are shown. [file 1477-7827-11-6-S3.tiff]

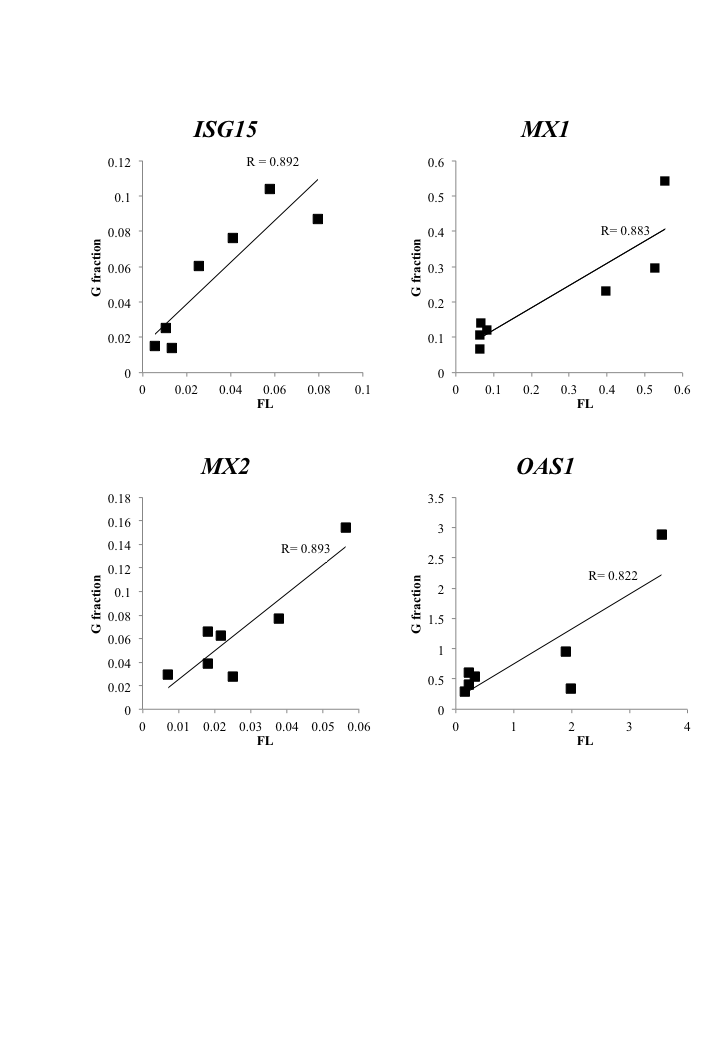

Supplement: Additional file 4 — Figure S2. Correlations between gene expression profiles in granulocyte fractions obtained by flow cytometry and Ficoll-Conray separation. Blood samples were divided and simultaneously analyzed using the two different methods. G: granulocyte, FL: flow cytometry. Data were normalized to GAPDH expression (n = 7). [file 1477-7827-11-6-S4.tiff]
